# Trial Sequential Analysis in systematic reviews with meta-analysis

**DOI:** 10.1186/s12874-017-0315-7

**Published:** 2017-03-06

**Authors:** Jørn Wetterslev, Janus Christian Jakobsen, Christian Gluud

**Affiliations:** 1 Copenhagen Trial Unit, Centre for Clinial Intervention Research, Dpt. 7812, Rigshospitalet, Copenhagen University Hospital, Blegdamsvej 9, DK-2100 Copenhagen, Denmark; 2 Centre for Research in Intensive Care, Rigshospitalet, Copenhagen University Hospital, Blegdamsvej 9, DK-2100 Copenhagen, Denmark; 3 Department of Cardiology, Holbæk Hospital, DK-4300 Holbæk, Denmark; 4 The Cochrane Hepato-Biliary Group, Copenhagen Trial Unit, Centre for Clinial Intervention Research, Dpt. 7812, Rigshospitalet, Copenhagen University Hospital, Blegdamsvej 9, DK-2100 Copenhagen, Denmark

**Keywords:** Meta-analysis, Random-effects model, Fixed-effect model, Interim analysis, Group sequential analysis, Trial sequential analysis, Heterogeneity, Diversity, Sample size, Information size

## Abstract

**Background:**

Most meta-analyses in systematic reviews, including Cochrane ones, do not have sufficient statistical power to detect or refute even large intervention effects. This is why a meta-analysis ought to be regarded as an interim analysis on its way towards a required information size. The results of the meta-analyses should relate the total number of randomised participants to the estimated required meta-analytic information size accounting for statistical diversity. When the number of participants and the corresponding number of trials in a meta-analysis are insufficient, the use of the traditional 95% confidence interval or the 5% statistical significance threshold will lead to too many false positive conclusions (type I errors) and too many false negative conclusions (type II errors).

**Methods:**

We developed a methodology for interpreting meta-analysis results, using generally accepted, valid evidence on how to adjust thresholds for significance in randomised clinical trials when the required sample size has not been reached.

**Results:**

The Lan-DeMets trial sequential monitoring boundaries in Trial Sequential Analysis offer adjusted confidence intervals and restricted thresholds for statistical significance when the diversity-adjusted required information size and the corresponding number of required trials for the meta-analysis have not been reached. Trial Sequential Analysis provides a frequentistic approach to control both type I and type II errors. We define the required information size and the corresponding number of required trials in a meta-analysis and the diversity (D^2^) measure of heterogeneity. We explain the reasons for using Trial Sequential Analysis of meta-analysis when the actual information size fails to reach the required information size. We present examples drawn from traditional meta-analyses using unadjusted naïve 95% confidence intervals and 5% thresholds for statistical significance. Spurious conclusions in systematic reviews with traditional meta-analyses can be reduced using Trial Sequential Analysis. Several empirical studies have demonstrated that the Trial Sequential Analysis provides better control of type I errors and of type II errors than the traditional naïve meta-analysis.

**Conclusions:**

Trial Sequential Analysis represents analysis of meta-analytic data, with transparent assumptions, and better control of type I and type II errors than the traditional meta-analysis using naïve unadjusted confidence intervals.

## Background

Most meta-analyses include too few randomised participants, to obtain sufficient statistical power that allow reliable assessment of even large anticipated intervention effects [1]. The credibility of statistical significant meta-analyses with too few participants is poor, and intervention effects are often spuriously overestimated (type I errors) or spuriously underestimated (type II errors) [2]. Meta-analyses of, e.g., cardiovascular, anaesthesiologic, and neonatal interventions have many false positive and false negative results, due to low statistical power in a meta-analysis when the required number of randomised participants or trials have not been reached [3–6]. Trial Sequential Analysis (TSA) of a meta-analysis may amend these problems [4, 7]. In this article, we aim to describe the origin, history, adaptation, and criticism of TSA.

Using TSA, we can handle a meta-analysis of several randomised clinical trials in an analogous manner to interim analysis of a single randomised clinical trial. If the accrued cumulative information fails to achieve the required number of randomised participants in order to detect or reject a specific assumed effect, the uncertainty of the estimate of the intervention effect will increase. The uncertainty will decrease the higher the fraction of the required information size the meta-analysis obtain. To statistically solve the problem with uncertainty, we expand the confidence interval, i.e., adjusting the threshold for statistical significance when the required information size has not been reached. The farther from the required number of randomised participants, the wider the confidence interval and the lower the statistical significance level needs to be in order to reliably assess the uncertainty of the point estimate.

**Fig. 1:**
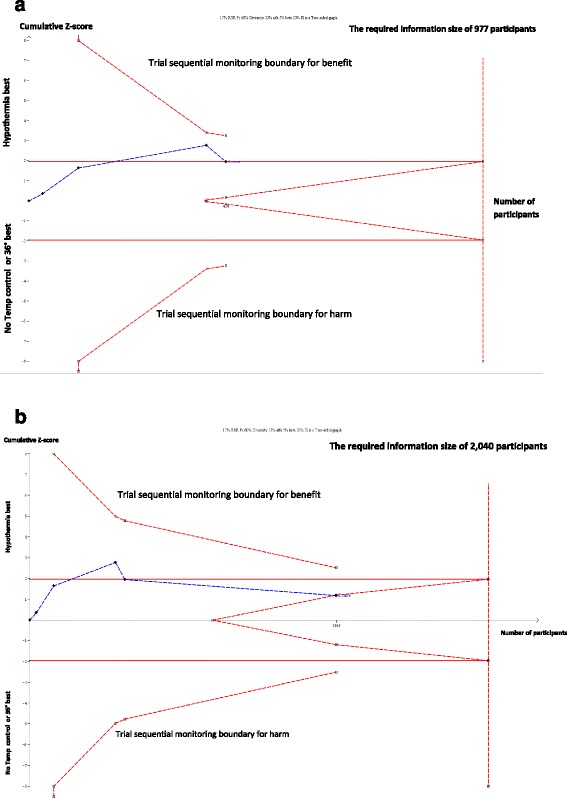
**a** Showing Trial Sequential Analysis of meta-analysis before the Target Temperature Management Trial. The *Z*-value is the test statistic and |*Z*| = 1.96 corresponds to a *P* = 0.05; the higher the *Z*-value, the lower the *P*-value. Trial Sequential Analysis (TSA) of mortality after out of hospital cardiac arrest patients, randomised to cooling to 33°–34 °C versus 36 °C or no temperature control in four trials performed before the Target Temperature Management (TTM) trial [16, 20]. The required information size to detect or reject the 17% relative risk reduction found in the random-effects model meta-analysis is calculated to 977 participants using the diversity found in the meta-analysis of 23%, mortality in the control groups of 60%, with a double sided α of 0.05 and a β of 0.20 (power of 80.0%). The cumulative *Z*-curve (black full line with quadratic indicatons of each trial) surpasses the traditional boundary for statistical significance during the third trial and touches the traditional boundary after the fourth trial (95% confidence interval: 0.70 to 1.00; *P* = 0.05). However, none of the trial sequential monitoring boundaries (etched curves above and below the traditional horizontal lines for statistical significance) have been surpassed in the TSA. Therefore, the result is inconclusive when adjusted for sequential testing on an accumulating number of participants and the fact that the required information size has not yet been achieved. The TSA-adjusted confidence interval is 0.63 to 1.12 after inclusion of the fourth trial [10, 12]. **b** showing Trial Sequential Analysis of meta-analysis after the Target Temperature Management Trial. The *Z*-value is the test statistic and |*Z*| = 1.96 corresponds to a *P* = 0.05; the higher the *Z*-value, the lower the *P*-value. Trial Sequential Analysis (TSA) of mortality after out of hospital cardiac arrest patients, randomised to cooling to 33°–34 °C versus 36 °C or no temperature control in five trials after inclusion of the Target Temperature Management (TTM) Trial [17]. The required information size to detect or reject the 17% relative risk reduction found in the random-effects model meta-analysis prior to the TTM Trial is calculated to 2040 participants using the diversity found in the meta-analysis of 65%, mortality in the control groups of 60%, with a double sided α of 0.05 and a β of 0.20 (power of 80.0%). The cumulative *Z*-curve (black full line with quadratic indicatons of each trial) touches the boundary for futility indicating that it will be unlikely to reach a statistical significant *P* < 0.05, even if we proceed to include trials randomising patients until the required information size of 2040 is reached. The result indicates that a 17% relative risk reduction (or more) may be excluded, even though the required information size has not been achieved, adjusting for sparse data and sequential testing on an accumulating number of patients [10, 12]

In TSA of a meta-analysis, we include the trials in chronological order and we handle the analysis of these trials as an interim analysis relative to the required number of randomised participants. TSA calculates the required number of participants, based on our predefined anticipated intervention effect, i.e., our alternative hypothesis [7–9]. The result of a trial sequential meta-analysis is displayed on a TSA diagram (e.g., Fig. 1a and b) with a TSA-adjusted confidence interval and an adjusted level of statistical significance, i.e, a lower threshold for statistical significance compared to the usual nominal of 0.05, if the required information size has not been reached [10].

In the above-mentioned adjustments, we take into consideration if the required number of randomised participants and corresponding trials, to show or reject a specific intervention effect, were reached or not. The required information size is defined as the number of participants and events necessary to detect or reject an *a priori* assumed intervention effect in a meta-analysis [11]. The required information size is not a single sample size, but a summation of sample sizes from a given number of included trials. Therefore, the calculation is performed considering the variability (heterogeneity variance) between the estimates of the intervention effects of the included trials.

In TSA, the sample size, required for a single randomised clinical trial to be conclusive for a specific intervention effect, is adjusted upward by an appropriate measure of the statistical heterogeneity in the meta-analysis in order to become the required information size. This is equivalent to using the variance in the random-effects model to calculate the required information size (the model variance based calculation of the required information size). In the TSA, we hereafter adjust the confidence interval of the point estimate and the threshold for statistical significance relative to the fraction of the required information size which has been accrued in the actual meta-analysis [11].

First, we will present a motivating example of a meta-analysis on hypothermia versus no hypothermia in comatose patients having survived cardiac arrest. Second, we present an updated meta-analysis with the results of a new trial, and we describe how this update has changed the conclusion of the preceding traditional meta-analysis. We also show how the use of TSA would appropriately have reduced the risk of a wrong conclusion in the first meta-analysis failing to achieve the required information size. Third, we shortly describe the historical development of sequential analyses in a single trial with interim analyses and in a cumulative meta-analysis of several trials. We explain how sequential meta-analysis can be performed with TSA [12]. Finally, we discuss the criticism that has been raised about TSA and we briefly describe the possibility for Bayesian meta-analysis as an alternative to both traditional naïve meta-analysis and TSA of a meta-analysis.

### A motivating example: how the Target Temperature Management-Trial changed the conclusion of the meta-analysis of trials with cooling of patients after out of hospital cardiac arrest

In TSA, we consider each interim-analysis result, produced after the addition of a new trial, a sequential meta-analysis. The possibility to include groups of several new trials at a time is, of course, also possible. This latter approach will decrease the number of interim-analyses in the cumulative meta-analysis [10]. However, updating the meta-analysis in a systematic review each time a new trial is published is a rational decision, and to update a systematic review before a new trial is initiated ought to become mandatory [13–15]. Previous trial results ought to be considered whenever we evaluate the cons and pros of designing new trials, as the evidence on a given intervention may already be sufficient [13–15]. It is surprising to see how little the TSA, conducted after each new trial has been interim-analysed, differs from the last TSA on groups of trials (e.g., TSA only updated every second year).

Figure 1 shows the result of a TSA of meta-analysis of four trials comparing a target temperature of 33°–34 °C versus no cooling, conducted before the initiation of the Target Temperature Management (TTM) Trial (Fig. 1a) [16–18]. The TSA shows that the four trials did not even reach half of the required information size to confirm or reject a 17% relative risk reduction which was the intervention effect indicated in a conventional meta-analysis of the trials [16]. The conventional confidence interval for the relative risk ratio of all-cause mortality in a traditional meta-analysis is 0.70 to 1.00 (*P* = 0.05), suggesting a reduction of mortality. The confidence interval and the *P*-value would not have been sufficient to claim a conclusive interim analysis stopping for benefit in a single randomised trial if analysed with Lan-DeMets’ group sequential monitoring boundaries [19]. For demonstrating a 17% relative risk reduction, the TSA-adjusted confidence interval of the relative risk is 0.63 to 1.12. This confidence interval shows that i) a target temperature of 33°–34 °C versus no cooling can either decrease or increase mortality, and ii) that definitive evidence has not yet been reached. The cumulative Z-curve in the figure does not pass through the trial sequential monitoring boundary for benefit; only the conventional and naïve *P* = 0.05 (Z = 1.96) level for a beneficial effect has been reached. Therefore, there is not sufficient information to document the effect, or there may not be a beneficial effect at all. Nevertheless, based on this evidence, international guidelines had recommended for ten years that the temperature of comatose cardiac arrest patients should be targeted to 33°–34 °C, calling the intervention »mild therapeutic hypothermia« [20]. No further randomised clinical trials of induced hypothermia versus no temperature control (or normothermia) in comatose cardiac arrest patients after resuscitation and admittance to intensive care units were conducted during this 10-year period. This may indicate that a *P*-value of 0.05 in the conventional meta-analysis was used as an unofficial »stopping boundary« for further trials within this same period.

In the TTM Trial, we compared the effect of cooling to target temperature 33 °C versus 36 °C on mortality of cardiac arrest patients [17, 18]. The updated TSA including the TTM Trial showed no statistically significant effect at the conventional level, as the Z-curve returned to the area with *P* > 0.05 (|Z| < 1.96) (Fig. 1b). Figure 1b shows that the cumulative Z-curve touches the futility-boundaries in the TSA diagram (see section ‘False Negative Meta-analyses’ below*)*. Therefore, the updated TSA indicates that a 17% relative risk reduction, or an even greater reduction, most likely can be rejected, although the pre-estimated required information size of 2040 patients has not yet been reached. It is not likely that a meta-analysis will ever show a 17% statistical significant relative risk reduction of mortality, even though the continued conduct of trials until a cumulated number of patients, corresponding to the required meta-analytic information size of 2040 patients, was reached (Fig. 1b). The conclusion is that hypothermia to 33°–34 °C does not seem to have a clinical important effect on mortality compared with no cooling or targeted normothermia (36 °C), as the 17% relative risk reduction only corresponds to a median of 3 weeks’ longer survival [17, 18]. Moreover, the original conventional meta-analysis before inclusion of the TTM Trial had a false positive result; the null hypothesis was falsely rejected. Whether the avoidance of fever is actually beneficial compared with no cooling at all, remains to be tested, as the TTM trial used cooling in both the intervention (target 33 °C) and the control group (target 36 °C).

### Interim-analyses during a randomised clinical trial with an accumulating number of participants

If a trial is stopped after an interim-analysis because of a *P* < 0.05 or the trial is continued if P ≥ 0.05, the real risk of committing a type I error will increase to more than 0.05 with the number of interim-analyses. Introducing an interim-analysis half-way in a randomised clinical trial, using a stopping *P*-value equal to 0.05 in both the half-way analysis and the final analysis, will increase the real maximal type I error risk to 8% [21, 22] (Table 1). If the procedure of interim analysis is performed as four interim analyses and one final analysis, with a constant level of statistical significance of 5%, the real type I error risk will be 14% [21]. A simulation study using repetitive testing on an accumulating number of participants in a single trial, has shown that the *P*-value will inevitably become less than 0.05, despite the true intervention effect being zero [23].

**Table 1:** Showing the level of cumulated type 1-error risk, if a threshold of 5% is applied constantly at each sequential significance testing, on an accumulating number of trial participants

Number of statistical significance tests	The cumulated type 1-error risk in %
1	5%
2	8%
5	14%
20	25%
100	37%
Infinitely many	100%

The resulting type 1-error risk will be larger than the nominal 5%, if a decisison is made to stop the inclusion of participants when *P* <0.05 and to continue when *P* ≥0.05 [22, 24]

A Bonferroni adjustment of the level of statistical significance, being 5% divided with the number of tests on accumulating data, assumes that all tests are conducted on independent data. As the tests on the accumulating trial population are not statistically independent, the Bonferroni-adjusted levels of statistical significance are most often too conservative [24]. The trial participants in an early sequential analysis are also included in the subsequent later sequential analyses. Therefore, there is an increasing overlap of trial participants included in the latest sequential analysis compared to participants included in the previous sequential analyses. The closer we come to the *a priori* calculated sample size, the Bonferroni adjustment becomes more and more unjustified (too conservative).

### Historical development of sequential analyses in a single trial with interim analyses

Methods to avoid an increased risk of a type I error due to repetitive testing on an increasing number of observations was described by Abraham Wald in 1945 in *Contributions to the theory of statistical estimation and testing hypotheses* [25]. Wald proposed »the sequential probability ratio test« in which the sequential testing continues until a definitive wanted or unwanted effect can be proved [26, 27]. According to this procedure, the trial continues as long as the results of the sequential tests fall within the so-called ‘zone of indifference’ amidst the two alternative hypotheses. This procedure, used as a quality assurance measure of production during the Second World War, has never achieved wide implementation in randomised clinical trials; possibly because the procedure is bound to continue infinitely as long as the true intervention effect lies between the two alternative hypotheses. Consequently, a decision to stop the trial may never become possible [28].

After the Second World War, Peter Armitage suggested more restrictive levels of statistical significance than 5% to stop a trial before the *a priori* calculated sample size was reached [21]. This procedure was applied in a number of interim analyses of large trials [29]. Furthermore, Stuart Pocock proposed a procedure in which the overall risk of type I error is limited to 5% by setting the statistical significance level to 0.05 divided by *k*, using *k-1* interim analyses and a final analysis [22]*.* This procedure is identical to the Bonferroni procedure for interim analyses and a final analysis of a single trial [30]. Researchers might find it peculiar to only declare statistical significance if *P* < (0.05/*k*), despite the estimated sample size has been reached and the required criterion for statistical independence was not fulfilled.

**Fig. 2:**
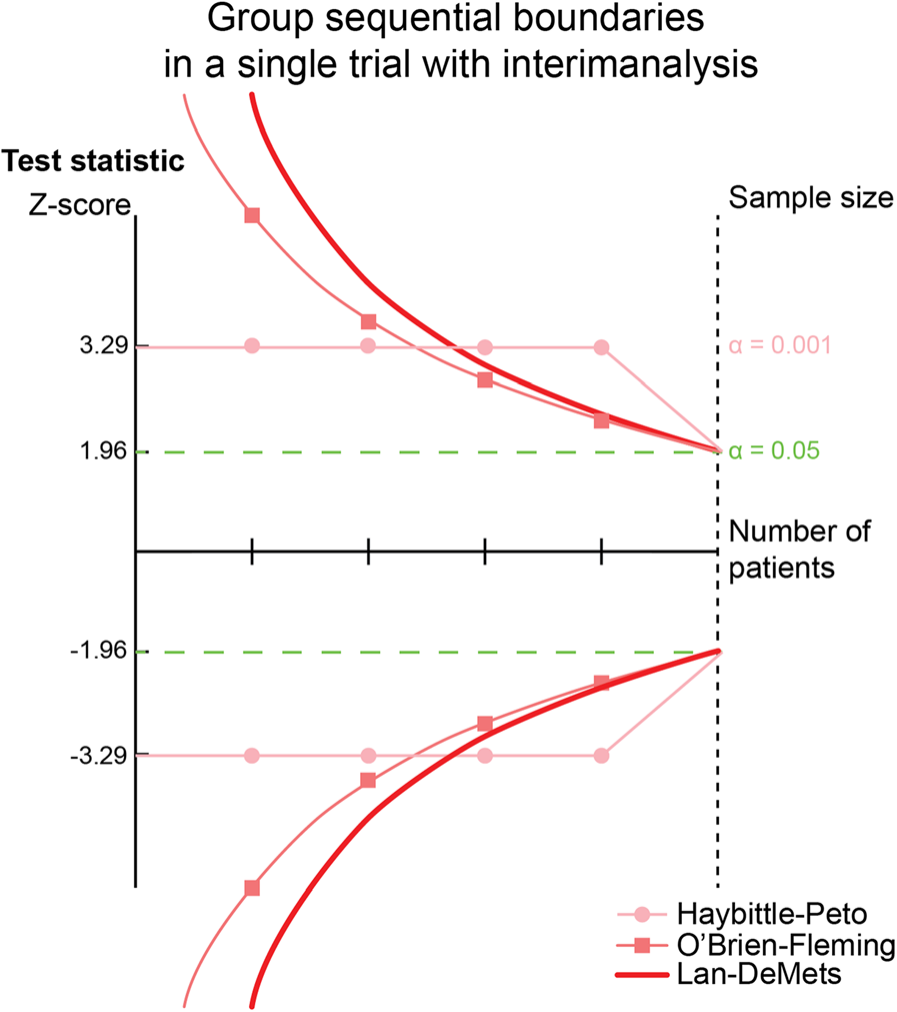
Showing three different group sequential boundaries in a single trial with interim analysis. The *Z*-value is the test statistic and a |*Z*| = 1.96 corresponds to *P* = 0.05; the higher the *Z*-value, the lower the *P*-value. This is a historical overview of group sequential boundaries for the cumulative *Z*-curve in relation to the number of randomised participant in a single trial [19, 32, 33]

In 1977, Richard Peto suggested the use of a maximal type I error risk (α-spending) in each of four interim analyses of 0.001 (1 promille) and 0.05 in the final analysis. As this would produce a summary additional type I error risk of 0.004 to the final 0.05, the total type I error risk would maximally be 5.4% [31] (Fig. 2). However, by a modest increase of the *a priori* estimated sample size, the summary maximal used type I error risk would remain within the usual 5%. As shown above, the rationale of statistical independence required for this procedure still lacks underlying reason as to why the trial participants in an early sequential analysis are also included in the subsequent sequential analysis.

In 1979, Peter O’Brien and Thomas Fleming proposed the group sequential design of trials with interim analyses, using exponential decreasing levels of statistical significance with the increasing number of patients in the sequentially analysed groups (Fig. 2) [32]. The recommendations of the International Conference on Harmonization – Good Clinical Practice, the U.S.A. Food and Drug Administration, and the European Medicines Agency on the design and analysis of randomised trials with interim analyses are mainly based on works from 1980s, primarily prepared by Gordon Lan, Kuyung Man Kim, and David DeMets (Fig. 2) [18, 33, 34]. Their works allow proper sequential testing at any time during the trial period, without unduly increasing the overall risk of a preset nominal type I error risk [34–36].

## Methods

### Avoiding the increased risk of random errors in randomised clinical trials with interim analyses

It is and should be mandatory to perform interim analyses in large randomised clinical trials addressing patient-centred outcomes. Even though the preplanned sample size has not been reached, thousands of patients might already have been randomised in a trial. Before we allow the trial to continue, there is a need to secure that no valid evidence showing superiority of one of the compared interventions exists. If one of the interventions (could also be placebo) with a sufficiently small uncertainty is superior to the other one in an interim analysis, it may be unethical to continue the trial. The explanation for this is that the superiority can be so large that it cannot be reversed even though we continue to randomise patients until the total, originally preplanned sample size is obtained. If the trial is continued despite the superiority of the intervention in one of the intervention groups, the patients in the other group will be exposed to an inferior (harmful) intervention and the trial must be stopped [37]. The use of interim analyses in a single randomised trial has to be planned at the design stage of the trial and protocolised upfront as group sequential analyses in the charter for interim analyses [33]. For the conduct of group sequential analyses, a sample size is calculated already at the design stage, based on the anticipation of a minimal important and realistic intervention effect of the primary outcome of the trial [36, 38] (see Appendix).

The sample size calculation considers the level of statistical significance at which we want to test a dichotomous or a continuous outcome when the full sample size has been reached. It is when the pre-calculated sample size has been reached, and only then, a two-sided *P*-value of less than 0.05, corresponding to a test-statistic Z-value of ±1.96, can be accepted as the statistical significance level when α has been set to 5% in the sample size calculation.

Interim analyses, with the potential to stop a randomised trial before the estimated (or fixed) sample size has been reached due to a positive, negative, or lack of the addressed effect, can be conducted for dichotomous and continuous outcomes by calculating the cumulative Z_*i*_-value at the *i*-th analysis (see Appendix). The calculated Z_*i*_-value is then related to the more restrictive level of statistical significance, the critical Z-value being the discrete group sequential boundary according to the actual accrued number of participants.

There is international consensus that the increase of type I error risk with sequential testing, including the risk of overestimating the intervention effect or underestimating the variance, at an interim analysis, should be outweighed by more restrictive levels of statistical significance before the *a priori* estimated (fixed) sample size has been reached [29, 31–37]. This is why ‘monitoring boundaries’, with significance levels much smaller than a nominal *P*-value of 0.05 (corresponding to much larger |Z|-values than ±1.96) are applied as criteria to stop a trial before achieving the estimated sample size [33].

Numerical integration is used to calculate the monitoring boundaries, being the critical levels of statistical significance for the Z_*i*_-values (and *P*-values) of the interim analyses [39]. Most often, the O’Brien-Fleming’s α-spending–function is applied and converted to sequential boundaries (critical values) for the Z_*i*_-values called Lan-DeMets’ sequential monitoring boundaries (Fig. 2) [18, 19]. The α-spending function allows only a small part of the total nominal type I error risk to be used initially in the sequential analyses, and with a modest increase of the estimated final (fixed) sample size, there is a full 5% type I error risk available for the final analysis when the *a priori* estimated sample size is reached. Lan-DeMets’ sequential boundaries allow testing whenever you want during the trial [34, 35]. If we plan, e.g., a half-way analysis in a randomised trial, we can monitor the *P*-value at this time point according to Lan-DeMets’ monitoring boundaries and suggest that the trial is stopped if the *P*-value is less than 0.003 which corresponds to a 99.7% confidence interval excluding 1.00 for a relative risk or 0.00 for a mean difference [34–36]. Therefore, sequential analyses become a theoretical decision tool to decide whether a trial should be stopped before the estimated (fixed) sample size is achieved, considering the sparse data and the repetitive testing during the trial [37].

### Avoiding the increased risk of random errors in cumulative meta-analyses with sparse data and multiple meta-analytic up-dates

The majority of meta-analyses include less than the required number of randomised participants and trials in order to become conclusive [1, 3, 5, 7]. There are two reasons for this. First, most randomised trials are underpowered [1, 3, 5, 7]. Second, the estimation of the required information size in a random-effects meta-analysis ought to incorporate the heterogeneity variance (between trial variance) [1, 7, 11]. Only 22% of the meta-analyses in The Cochrane Library have 80% power to conclude whether there is an intervention effect of 30% or not when the usual maximal risks of type I error (α) of 5% and type II error (β) of 20% are applied [1]. This lack of power is primarily caused by small trials and a considerable heterogeneity variance between the estimates of the intervention effect in the included trials [1].

Meta-analyses can be conducted with a fixed-effect model or a random-effects-model [40, 41]. In the fixed-effect model, we assume one true underlying effect in all the included trials. In the random-effects model, we assume that the true underlying effects vary from trial to trial according to a normal or log normal distribution. Often, the fixed-effect assumption is unrealistic as the possible underlying effect may depend on, e.g., doses of a pharmacological intervention, duration of the interventions, timing of the outcome assessment, and differences between the trial populations. These differences between the included trials are called clinical heterogeneity. Due to these factors and possibly random variation, the included effect estimates often show considerable variation defined as statistical heterogeneity and measured as large inconsistency (I^2^) [42] and large diversity (D^2^) [11]. Considerable statistical heterogeneity leads to increased uncertainty, expressed as a wider confidence interval of the intervention effect when the meta-analytic estimate is calculated in a random-effects model. Early meta-analyses conducted before the required information size and the corresponding number of trials are achieved [43], often wrongly show unrealistic large intervention effects as well as statistical significance which cannot be reproduced when the amount of required information is adequately considered [44, 45]. The reliability in early meta-analyses is lower compared to their updated counterparts years later [2]; the estimated intervention effects, when further trials are included in the meta-analysis update, become considerably lower than previously estimated [2].

**Fig. 3:**
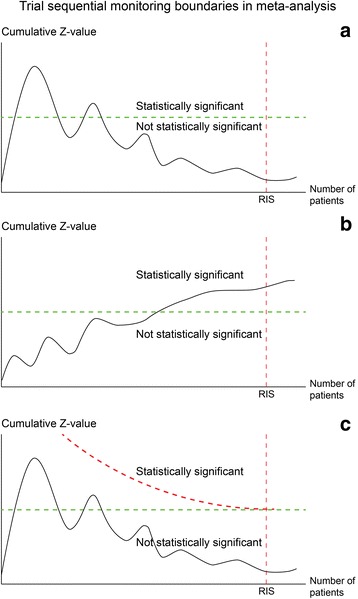
Showing trial sequential monitoring boundaries for benefit and harm in a cumulative meta-analysis. The *Z*-value is the test statistic and |*Z*| = 1.96 corresponds to *P* = 0.05; the higher *Z*-values, the lower the *P*-values. **a** Shows how an early statistical significance no longer is present in a cumulative meta-analysis when the required information size has been reached. **b** Shows how an early lack of statistical significance emerges later when the requiered information size is achieved. **c** Shows how an early statistical significance can be avoided by adjusting the level of statistical significance. The etched upper curve is the group sequential boundary adjusting the level of statistical significance for multiple testing and sparse data. *Z*-value is shown on the y-axis and on the x-axis *IS* is the required information size [10]

A large simulation study of random-effects meta-analyses shows that there is a considerable risk of overestimating the intervention effect when the required information size has not been reached [6]. These results were based on the assumption that the ‘true’ intervention effect was zero while the frequencies of events in the control groups and the heterogeneity variance were assumed similar to those in large cardiologic meta-analyses [6]. It has been shown empirically that approximately 25% of cardiologic meta-analyses are inconclusive because of lack of power [3]. Turner and colleagues showed that the trials and the meta-analyses of Cochrane systematic reviews have limited power [1]. In Cochrane meta-analyses, each total number of analysed participants provide only 22% of the meta-analyses with an 80% power to detect or refute a 30% relative risk reduction (which is a large intervention effect) [1] (Fig. 3). Recently, Imberger and colleaques confirmed these results in meta-analyses of anaesthesiological interventions [46]. Accordingly, four out of five meta-analyses did not have the statistical power to address even substantial intervention effects. The number of meta-analyses with sufficient power to address smaller and clinically more plausible intervention effects are, undoubtedly, even smaller.

If we test with a constant level of statistical significance (e.g., 5%) on the way towards the required information size, the risk of type I error is increased to more than 5%. The problem for cumulative meta-analyses, due to repeated updating and consecutive calculation of 95% confidence intervals, with inclusion of results from new randomised trials is, therefore, analogous to interim analyses of a single trial [8, 9]. Thus, we, as well as others, recommend that the interpretation of meta-analyses in systematic reviews is done alongside with a sequential analysis, e.g., Trial Sequential Analysis (TSA) [46, 47]. The purpose of using TSA is to avoid the risk of type I and type II errors due to sequential testing on a constant statistical significance level and with inclusion of fewer participants than the required number in order to detect or reject specified effects [7, 10, 11]. It is possible to accommodate Gordon Lan and David DeMets’ group sequential analysis for interim analysis in a single randomised trial to the updating of cumulative meta-analysis as it progresses with the addition of trials. This is done with an appropriate continuous use of type I error risk and an α-spending function of the allowed total nominal type I error risk, so that when the required information size and the required number of trials have been reached and beyond, the risk is kept below 5%. The trial sequential monitoring boundaries generated this way make it possible to test if significance is reached and to adjust the confidence intervals every time new trials are added to the meta-analysis. The latter is a prerequisite for using sequential boundaries in cumulative meta-analyses of trials with varying sample sizes [10, 12].

Besides applying the observed estimate of statistical heterogeneity—the observed statistical diversity (D^2^) [11, 41] in the most recently conducted meta-analysis—it may be reasonable to apply an expected heterogeneity in the calculation of the required information size, especially when the observed heterogeneity is zero [48]. As it is unlikely that diversity will stay zero when larger trials are added, an expected heterogeneity may be used in a sensitivity analysis (e.g., a diversity of 25% or the upper confidence interval of the I^2^ (provided by the TSA program)) when the required information size is calculated [48, 49]. It may also be wise in a *post hoc* calculation of the required information size to apply the least likely intervention effect, i.e., the confidence limit of the summary estimate in the meta-analysis confidence interval closest to the null effect. The latter is a conservative approach facilitating the evaluation of whether a meta-analysis may show an effect of the least likely magnitude in a TSA. If a TSA with such an approach shows a statistical significant intervention effect, judged by the TSA-adjusted confidence interval, there is a very high probability that the intervention has an effect, provided that the included trials are at low risk of bias. In contrast, there will only be very low evidence of effect if the TSA-adjusted confidence interval does not exclude the null effect for an intervention effect of a magnitude indicated by the point estimate.

## Results

### False positive meta-analyses

It is necessary to assume or address a specific magnitude of the intervention effect, different from zero, in order to calculate the sample size in a single trial. Therefore, when a sample size is estimated, we relate not only to the null hypothesis but also to a specific alternative hypothesis. The alternative hypothesis is the assumption or the anticipation of a specific magnitude of the intervention effect different from zero. Most often random-effects meta-analysis will be the preferred appropriate method to estimate the precision weighted average effect as it does not ignore the statistical heterogeneity variance. If statistical heterogeneity is anticipated, the information size in the conclusive meta-analysis ought to be an upward adjusted sample size of a corresponding adequately powered single trial. The upward adjustment is done with the variance expansion shifting from a ‘fixed-effect’ model to a ‘random-effects’ model, see Appendix [11].

The described example from cooling of patients after out of hospital cardiac arrest is far from being unique (Fig. 1). Among meta-analyses of interventions for neonatal patients, there were approximately 25% to 30% false positive results [5, 50]. In 2009, we showed empirically that the use of Lan-DeMets’ trial sequential monitoring boundaries eliminated 25% of the false positive traditional interim-meta-analyses. This analysis included 33 final meta-analyses with sufficient information size to detect or reject a 15% relative risk reduction [44]. In 2013, we showed that 17% of cardiovascular meta-analyses with *P* < 0.05 were most likely false positive [3]. In 2015, we showed that less than 12% of meta-analyses of anaesthesiological interventions had 80% power to show a 20% relative risk reduction [46].

There may be other important reasons for a traditional meta-analysis to yield a false positive result than only the increased risk of random errors. A risk of systematic error (bias) in the included trials is a frequent cause of overestimation of benefit and underestimation of harm – sequential meta-analyses do not in any way solve problems with bias [51–58]. Therefore, it is recommended that every single trial included in a systematic review with meta-analysis be evaluated for risks of bias. This evaluation should encompass the following domains: generation of the allocation sequence, allocation concealment, blinding of patients and caregivers, blinding of outcome assessment, report on attrition during the trial, report on outcomes, and industry funding. Other types of bias may also need to be considered [51–58].

### False negative meta-analyses

**Fig. 4:**
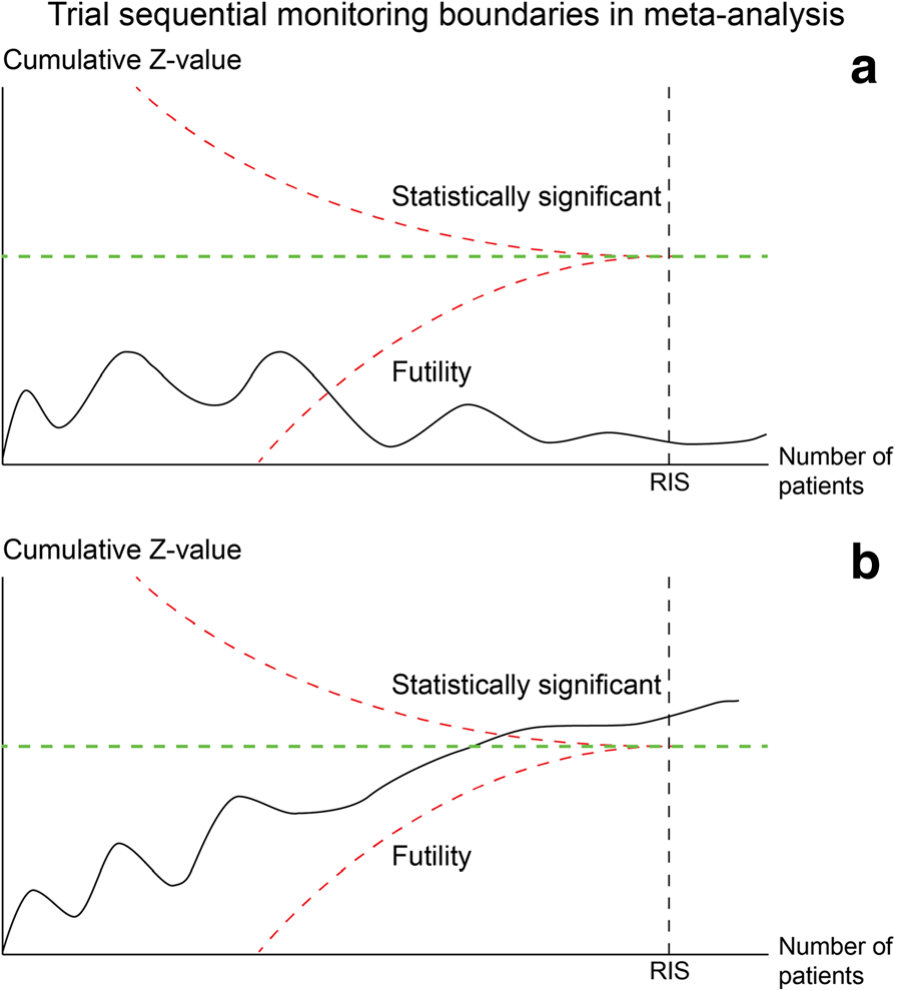
Showing trial sequential monitoring boundaries for benefit and futility in cumulative meta-analysis. The Z-value is the test statistic and |*Z*| = 1.96 corresponds to *P* = 0.05; the higher Z-values, the lower *P*-values. **a** Shows how trial sequential monitoring of a cumulative meta-analysis, before the requiered information size (*IS*) is achieved, makes it likely that the assumed effect is in fact absent when the Z-curve surpasses the futility-boundary (etched curve). **b** Shows how trial sequential monitoring of a cumulative meta-analysis, before the required information size (*RIS*) is achieved, makes it likely that the assumed effect is in fact true when the Z-curve surpasses the trial sequential monitoring boundary for benefit (etched curve). Lan-DeMets’ α-spending-function has been applied for the construction of the trial sequential monitoring boundaries, the critical Z-values [10]

Lack of a statistical significant intervention effect in a traditional meta-analysis is not necessarily evidence of no effect of the intervention. »Absence of evidence is not evidence of absence of effect« [59]. Nevertheless, sequential meta-analyses with the TSA software may show that the meta-analysis has sufficient statistical power to reject an intervention effect of a specific magnitude even though the estimated required information size has not yet been reached (Fig. 4).

This can be done by calculating the non-superior and non-inferior trial sequential monitoring boundaries, the socalled ‘futility boundaries’. Futility boundaries indicate when the assumed effect could be considered unachievable. Futility-boundaries are calculated using a power function analogous to the α-spending function for constructing superiority- and inferiority-boundaries with application of numerical integration [36]. The example with cooling of comatose patients after cardiac arrest shows a situation where the assumed intervention effect of 17% relative risk reduction can be rejected because the Z-curve crosses the futility-boundary (Fig. 1b). However, this is not always what happens. We found that in 25 of 56 (45%) published cardiovascular systematic reviews in The Cochrane Library, the actual accrued information size failed to reach what was required to refute a 25% relative risk reduction [3]. Only 12 of these reviews (48%) were recognised as inconclusive by the authors. Of the 33 meta-analyses not showing statistical significance, only 12 (36%) were truly negative in the sense that they were able to reject a 25% relative risk reduction [3]. This illustrates that the statistical power is also low in many cardiovascular meta-analyses, and false conclusions are imminent. Within other medical specialities, the problems are likely to be even bigger as trials and meta-analyses usually include less patients. Nevertheless, sequential meta-analyses with calculated futility-boundaries may, in some instances, contribute to adequately declare the *a priori* anticipated intervention effect to be unachievable, though the required information size was not reached [10].

Analogous to the false positive meta-analyses, a meta-analysis may result in a false negative result due to bias. Bias is a frequent cause for underestimation of harmful intervention effects [51–57], and therefore, the preliminary defined bias risk domains should also be evaluated for all included trials when it comes to serious and non-serious adverse events [51–58].

## Discussion

We have explained and shown how the use of TSA may assist the meta-analyst in controlling risks of type I and type II errors when conducting meta-analyses. The use of TSA has now increasingly been advocated by authors, both inside and outside The Cochrane Collaboration [47, 60, 61]. However, the use of TSA is not easy, may be misused, and has been critisised [62].

If TSA is designed and conducted after data were collected, there is a danger that the analysis becomes data driven and that it may not be sufficiently stringent to address a predefined alternative hypothesis [63–65]. However, using data-driven hypotheses and analyses is a critique that could potentially be directed against all meta-analyses. This is why, for each TSA, the anticipated intervention effect, the anticipated between trial heterogeneity, and the proportion of the outcome in the control group, should be part of a peer reviewed protocol, published prior to the conduct of the systematic review and the TSA [49, 64, 65]. These considerations should also impact the choice of the meta-analytic model, e.g., whether to give most credibility to the fixed-effect or the random-effects model and how to calculate the required information size [11, 65].

TSA has been criticised for transferring a method from a decision theoretical universe in a single randomised clinical trial into a universe where the result does not directly impact the subsequent decisions [63–66]. The postulate seems to be that no matter that a TSA shows benefit, harm, or lack of a relevant effect, it will not impact any part of the already finalised trials, and possibly, not decisions to stop or continue ongoing trials, or to initiate trials. This point of view seems to unduly emphasise the difference between the consequences of an interim-analysis in a single trial and the consequences of a sequential meta-analysis of several trials. First, the systematic review is placed at the top of the generally recognised hierarchy of evidence, meaning that the systematic review is considered the most likely reliable source of evidence, implicating whether an intervention should be implemented in clinical practice or further trials should be launched [52, 53]. Interventions are often recommended in clinical guidelines and implemented in clinical practice when a meta-analysis shows statistical significance on the traditional naïve level (*P* < 0.05) [16, 18, 67–69]. Furthermore, the chance that a meta-analysis is updated in The Cochrane Library is apparently 57% higher when P ≥ 0.05 than when *P* < 0.05 [4, 45]. This indicates that meta-analyses with *P* < 0.05 contribute to the decision to stop doing further trials or to decide if meta-analyses should be updated or not.

Critics of sequential meta-analysis have stressed that the method emphasises too heavily the result of the statistical significance test instead of the 95% confidence interval [70]. However, the fundamental problem is not whether the result is presented as a *P*-value or as a confidence interval, but it is foremost because a (1-α)% confidence interval is based upon the choice of the maximally allowed type I error risk (α). If we use naïve unadjusted confidence intervals when the required information size is still not reached, we will be led to make hasty and false declarations of statistical significant effects, likely to be refuted if further trials are added. With TSA we adjust the confidence interval for the incomplete meta-analytic information size and for multiple testing [4]. It has been claimed that a traditional 95% confidence interval is sufficient to evaluate whether the intervention works or not [70], but the traditional 95% confidence interval exclusively relates to the null hypothesis and not to a relevant alternative hypothesis [68, 71]. Thereby, the supporters of the traditional confidence interval forget that the rejection of the null hypothesis (the conventional 95% confidence interval excluding the null effect), does not in itself lead to the acceptance of a relevant alternative hypothesis [71]. Premature rejection of the null hypothesis, in the case of sparse data, may be dismissed if these data become sufficient to conclude on a specific alternative intervention effect that is different from the null hypothesis.

A traditional unadjusted 95% confidence interval excluding the null effect and accepting an effect indicated by, e.g., the point estimate, is sufficient as a criterion for statistical significance only when the required information size has been reached. If the number of randomised participants in the meta-analysed trials is less than the required, the confidence interval needs to be adjusted [34, 36]. By exclusively applying a 95% confidence interval in a meta-analysis, one does not automatically account for the lack of required power in the meta-analysis to conclude on an effect size indicated by, e.g., the point estimate [71]. Therefore, in relation to a relevant and realistic alternative hypothesis, the traditional unadjusted confidence interval will represent a too narrow confidence interval which by chance does not include the null effect, and accordingly, the observed effect of the intervention may be misleading [71, 72]. The credibility of the traditional confidence interval relies on the fact that the required information size for a specific effect has been achieved, and thereby, the ability to conclude on an alternative hypothesis [59, 63–65].

TSA has also been criticised for being a too conservative approach as one may decide to use a too sceptical *a priori* intervention effect and use the total variance in the random-effects meta-analysis to calculate the required information size. The use of an *a priori* intervention effect does not consider the intervention effect estimated from the data already accrued; however, applying such an approach may in fact lead to even larger required information sizes [73]. Moreover, to think of the total variance in the random-effects model as a result of random variation alone, could be seen as a ‘worst-case scenario’ of risk of random error [73]. However, we may rarely know when a variation is caused by systematic differences or by random variations [52]. Therefore, it seems mandatory to perform an analysis, assuming that all the variance encountered in the random-effects meta-analysis is arising from ‘play of chance’ [46, 47].

Elena Kulinskaya and John Wood [43] argued, in their important article from 2013, that when estimating the information size in random-effects model meta-analyses, it is too simplistic to just increase the required information size with the variance increase, going from a fixed-effect to a random-effects model. Kulinskaya and Wood [43] persuasively showed that the necessary number of future trials to be included should be given with a lower limit (i.e., minimal number), regardless of the sample sizes of the trials, before the power of the random-effects model meta-analysis becomes sufficient to detect or reject a prespecified clinically relevant intervention effect. Kulinskaya and Wood also showed that increasing the number of future trials in a random-effects model meta-analysis might decrease the required information size estimated for additional future trials to render sufficient power of the random-effects meta-analysis [43]. We welcome the proposals for modifying the plan on number of subsequently included trials and their sample size. These considerations are in line with the findings of Joanna in’t Hout et al. [74], Alexander Sutton et al. [73], Jeffrey Valentine et al. [75], and Michael Borenstein et al. [76]. However, we would still argue that the difference between the required information size and the accrued information, may contribute importantly to the estimation of the necessary sample size in future trials, especially if coupled with the considerations proposed by Kulinskaya and Wood [43]. If we use the weighted estimate of the variance in previous trials as being the best estimate of the variance for the future trials, we may need 50% (Appendix) more trials than the minimal number required to cover the information gap of the required minus the acquired information size (*RIS-AIS*) (Appendix). Following an example given by Kulinskaya and Wood [43], we will be able to cover the information gap suggested by *RIS-AIS* with 12 trials instead of the minimal required number of eight trials. As outlined by Kulinskaya and Wood, we would be able to further decrease the total number of future randomised patients by increasing the number of future planned trials even more. However, this will be at the expense of dramatically decreasing the power of each new trial to detect the difference, indicated so far by the point estimate of the meta-analysis (or even the minimal important difference). Certainly, we could choose to increase the number of future trials with only one or two. However, the corresponding information size will still be huge. The minimal required number of trials calculated as the first integer greater than *c* ⋅ *τ* ^2^ (where *c* is a figure relating to the information already gained and *τ* ^2^ is the between trial variance, Appendix), and the corresponding meta-analytic information size, may be optimal because it provides each of the new, equally sized, trials with the same power as the ‘planned’ random-effects meta-analysis aimed to detect or reject a similar intervention effect. However, for most interventions, these huge trials will be unrealistically large to conduct. Alternatively, increasing the number of trials corresponding to a required extra information size of *RIS-AIS* will still provide such trials with a power of 80% to detect or reject an intervention effect of 2.5 times the effect indicated in the meta-analysis. Increasing the number of trials even further than the number corresponding to *RIS-AIS* will decrease the power of these trials with approximately 10% per additional trial (or increase the detectable alternative to three times or more the effect indicated in the meta-analysis). Such trials will subsequently be substantially underpowered to detect or reject even much larger intervention effects than the realistic difference, or even the minimal important difference. This will obviously destroy the integrity of such small future trials and they will generally, and rightfully so, be disregarded as heavily influenced by random error (‘play of chance’). Therefore, the *RIS* and thereby the *RIS-AIS* seem to be a fair trade-off between the number of required additional randomised participants and the number of required additional trials. In two examples given by Kulinskaya and Wood, the number of additional randomised participants is reduced from 4700 to 720 and from 11,200,000 to 300,000 when using *RIS-AIS* at the expense of four more trials than the minimal number of trials required. However, we agree, that a reasonable strategy for resolving the question of the presence or absence of a specific intervention effect with an adequately powered random-effects model may include a first trial with a sample size equal to the sample size indicated by formula 1 in the Appendix. This is a sample size corresponding to the minimal number of required trials. Such a trial may very well be substantially larger than the total acquired information size in the meta-analysis conducted before the trial. When the result from such a trial becomes available, the updated cumulative meta-analysis using the *a priori* anticipated intervention effect and a new estimate of the between trial variance may be used in a fixed-effect or a random-effects model to evaluate how far we will be from a conclusion of whether the intervention effect exists or not. The fixed-effect model may then turn out to be the most appropriate model to evaluate the pooled intervention effect when one or a few trials heavily dominate the entire accumulated evidence [77].

Nevertheless, we must be aware that including new trials in a cumulative meta-analysis may change the estimate of the ‘between trials variance’ as well as the proportion of events in the control group which are both essential for estimating the required information size and the corresponding number of required future trials. If diversity and the proportion of events in the control group change substantially, the magnitude of the required information size and the corresponding number of required future trials may change accordingly. This is the phenomenon of the ‘moving target’ which critics hold against TSA. However, a moving target seems better than having no target at all. Recently, we documented that in prospective application of TSA in very large cumulative meta-analyses, TSA prevented false positive conclusions in 13 out of 14 meta-analyses when *RIS* was not reached [45].

### Trial Sequential Analysis: a position between frequentist and Bayesian thinking

TSA of meta-analysis like the sequential analysis of a single randomised trial, originates from frequentist statistics [29]. The frequentist way of thinking was initially based on testing of the null hypothesis. This applies to both the *P*-value and its relation to an a priori accepted maximal type I error risk (α) and the possibility of including a null effect in the corresponding (1-α)% confidence interval [29]. The anticipation of an intervention effect of a specific magnitude, the alternative hypothesis, and subsequently the calculation of a required information size enabling the conclusion whether such an effect could be accepted or rejected, is, however, intimately related to the Bayesian prior.

TSA contains an element of Bayesian thinking by relating the result of a meta-analysis to the *a priori* point estimate of the intervention effect addressed in the analysis [77]. Bayes’ factor (BF) for a trial result is the ratio between the probability that the trial data originates under the null hypothesis, and the probability that the trial data originates under the alternative hypothesis or even several alternative hypotheses [72, 78, 79]. The posterior odds ratio for the estimate of the intervention effect after a new trial is added is calculated given the prior odds ratio for the intervention effect before the trial as: posterior odds ratio = BF x prior odds ratio [79]. In a Bayesian analysis, the prior takes form of an anticipated probability distribution of one or more possible alternative hypotheses or intervention effects which multiplied with the likelihood of the trial, results in a posterior distribution [79].

A methodological position between the frequentist and the Bayesian thinking can be perceived both in sequential interim-analyses of a single trial and in TSA of several trials [29]. Both have a decisive anticipation of a realistic intervention effect, although a full Bayesian analysis should incorporate multiple prior distributions with different anticipated distributions of intervention effects: e.g., a sceptical, a realistic, and an optimistic prior [79]. The TSA prioritise one or a few specific alternative hypotheses, specified by point estimates of the anticipated effect in the calculation of the required information size just as in the sample size estimation of a single trial [11].

The incentive to use sequential analyses arise because the true effect is not known and the observed intervention effect may be larger than the effect addressed in the sample size estimation of a single trial as well as in the estimation of the required information size for a meta-analysis of several trials. The need to discover an early, but greater effect than the one anticipated in the sample or information size calculation, or to discard it, thereby originates. If the intervention effect, in relation to its variance, happens to be much larger during the trial or the cumulative meta-analysis, this will be discovered through the breakthrough of the sequential boundary. However, this may also be problematic as too small sample sizes (in relation to the true effect), as mentioned, increase the risk of overestimation of the intervention effect or the risk of underestimation of the variance. In other words, due to a factitious too small sample size, we may erroneously confirm an unrealistic large anticipated intervention effect due to the play of chance.

There seems to be an ancestry between the sceptical prior in a Bayesian analysis and the use of a realistic intervention effect in a sequential analysis when the sample size in a single trial or the information size in a meta-analysis should be calculated [77, 78]. The smaller the effect, the greater the demand for quantity of information, and the sequential statistical significance boundaries become more restrictive. In other words, it becomes more difficult to declare an intervention effective or ineffective, in case the required information size is not achieved.

Christopher Jennison and Bruce Turnbull, however, have shown that on average, when a small, but realistic and important intervention effect is anticipated, a group sequential design requires fewer patients than an adaptive design, e.g., re-estimating the (fixed) sample size after the first interim analysis [80]. The group sequential design seems more efficient than the adaptive design. In line with mathematical theory [72], simulation studies [6], and empirical considerations [44, 45, 81, 82], there is evidence that small trials and small meta-analyses by chance tend to overestimate the intervention effect or underestimate the variance. Early indicated large intervention effects are often contradicted in later published large trials or large meta-analyses [6, 45, 81, 82]. The reason might be that statistical confidence intervals and significance tests, relating exclusively to the null hypothesis, ignore the necessity of a sufficiently large number of observations to assess realistic or minimally important intervention effects. The early statistical significance, at the 5% level, may be a result of an early overestimation of the intervention effect or an underestimation of the variance, or both, when the required information size for a realistic effect is not achieved. In general, it is easier to reject the null hypothesis than to reject a small, but realistic and still important, alternative hypothesis [64]. The null hypothesis can never be proven, and in practice, this means that it can never be completely discarded, as this would require an infinitely large number of observations.

The reason for early spurious significant findings may be quite simple, although not self-evident. Even adequate randomisation in a small trial lacks ability to ensure the balance between all the involved, known or unknown, prognostic factors in the intervention groups [81]. When we find a statistically significant intervention effect in a small trial or in a small meta-analysis, it is often due to insufficient balance of important prognostic factors, known or unknown, between the intervention groups. Therefore, it is not necessarily intervention effects that we observe, but rather an uneven distribution of important prognostic factors between groups. In addition to the described risks of random error, the overall risk of bias which includes the risk of publication bias makes it understandable why published trials and meta-analyses often result in unreliable estimates of intervention effects [2, 83].

The power of frequentist inference in a single trial and in a meta-analysis of several trials lies in two basic assumptions. First, the only decisive difference between the intervention groups during the trial is the difference between the interventions. We conclude that ‘despite everything else’, the measured difference in the outcome is due to different properties of the interventions because everything else seems equal in the groups. In a small trial and a small meta-analysis, the assumption, that all other risk factors are equally distributed in the two intervention groups, may not be fulfilled as described above, even though adequate bias control has been exercised. Second, the power of frequentist inference depends on the correctness of applying the ‘reverse law of implication’ from mathematical logic (see Appendix): that a sufficiently small *P*-value, calculated as the probability that we got a specific trial result when the null hypothesis is in fact true, leads us to discard the null hypothesis itself. This assumption, which never totally excludes the possibility that the result of a trial may agree with or be a result of the null hypothesis, demands a specific *a priori* chosen threshold for statistical significance. That is, a sufficiently small *P*-value leads us to regard the trial result as virtually impossible under the null hypothesis, and, therefore, we regard the opposite to be true and discard the null hypothesis. This automatically raises the question: how small a *P*-value should be before we can apply the ‘reverse law of implication’. Or alternatively expressed, does a *P*-value less than an *a priori* chosen threshold of statistical significance reject the null hypothesis? Ronald A. Fisher, already in 1956, warned against using a statistical significance level of 5% in all situations [84]. Nevertheless, ever since, it seems to have broadly been implemented as a criterion for conclusion in medical research [83], and this is likely wrong [85].

Sequential interim-analyses in a single trial and TSA of meta-analyses of several trials deal systematically and rationally with the misunderstood application of a constant level of statistical significance (*P* < 0.05), unrelated to the accrued fraction of the pre-calculated required (fixed) sample or information size and number of trials.

## Conclusions

Most systematic reviews with meta-analyses, including Cochrane systematic reviews, do not have sufficient statistical power to detect or reject even large intervention effects. Meta-analyses are updated continuously, and, therefore, ought to be regarded as interim-analyses on the way towards a required information size. The evaluation of meta-analyses ought to relate the total number of randomised participants to the required meta-analytic information size and the corresponding number of required trials considering statistical diversity. When the number of participants in a meta-analysis is less than the required, based on a realistic and minimally important intervention effect, the constant application of a traditional naïve 95% confidence interval or a naïve 5% statistical significance threshold will lead to too many false positive and false negative conclusions. The Lan-DeMets’ sequential monitoring boundaries in TSA offer adjusted, expanded confidence intervals and adjusted, restrictive thresholds for statistical significance when the diversity-adjusted required information size and the required number of trials for the meta-analysis has not been reached. A Bayesian meta-analysis, using prior distributions for both the intervention effect and the statistical heterogeneity, may even be more reliable for deciding whether an intervention effect is present or not. However, the Bayesian meta-analysis also poses difficulties with interpretation. Until easy-to-use software programs for full Bayesian meta-analysis become accessible, TSA represents a better assumption-transparent analysis than the use of traditional meta-analysis with unadjusted confidence intervals and unadjusted thresholds for statistical significance.

## Appendix

### Sample size in a single randomised trial

#### Dichotomous outcome

When the intervention and the control group are equal-sized groups, the sample size (SS) is calculated as [38]:

$$ S S=4\cdot {\left({Z}_{\alpha /2}+{Z}_{\beta}\right)}^2\cdot \frac{v}{\theta^2},\kern0.5em  w i t h\kern0.5em  v= P\cdot \left(1- P\right)\kern0.5em  o g\kern0.5em  P=\left({P}_E+{P}_C\right)/2 $$

In this formula, *P*_*E*_ and *P*_*C*_ are the frequencies of the outcome in the experimental group and the control group, *ϴ* is the intervention effect that the trial wants to address with *μ = P*_*C*_ *– P*_*E*_, α and β are the maximally allowed type I and type II error risks, and ν is the variance of the outcome difference between the two groups.

The test statistic *Z*_*i*_ at the *i-*th interim analysis for the dichotomous outcome measure:

$$ {Z}_i=\frac{P_{Ei}-{P}_{Ci}}{\ \sqrt{Var\left({P}_{Ei}-{P}_{Ci}\right)}} $$

with *P*_*Ei*_ − *P*_*Ci*_ being the estimate of the intervention effect at the i*-*th interim analysis, and *Var*(*P*_*Ei*_ − *P*_*Ci*_) is the variance of the estimate [36].

#### Continuous outcome

If the anticipated difference between the means in the control group and the experimental group is assumed to be *X*_1_–*X*_2_ with the standard deviation *SD*, α and β being the type I and type II error risks, the *SS* is calculated as [38]:

$$ S S=4\cdot {\left({Z_{1-}}_{\alpha /2}+{Z}_{\beta}\right)}^2\cdot \frac{{\left({\mathrm{X}}_1{\textstyle \hbox{-} }{\mathrm{X}}_2\right)}^2}{{\mathrm{SD}}^2} $$

The cumulative *Z*_*i*_-value at the *i*-th interim analysis is calculated as:

$$ {\mathrm{Z}}_{\mathrm{i}}=\frac{{\mathrm{X}}_1{\textstyle \hbox{-} }{\mathrm{X}}_2}{\mathrm{SD}\left({\mathrm{X}}_1{\textstyle \hbox{-} }{\mathrm{X}}_2\right)} $$

*X*_*1i*_ *– X*_*2i*_ being the estimate of the intervention effect at the *i*-th interim analysis, and *SD*_*(X1i –X2i)*_ its standard deviation [36].

### The required information size in a meta-analysis

The required information size (*RIS*) in a meta-analysis of a dichotomous outcome can be calculated as (see the definition of μ and ν above) [11]:

$$ R I S=\frac{1}{1-{\mathrm{D}}^2}\cdotp 4\cdotp {\left({Z_{1-}}_{\alpha /2}+{Z}_{\beta}\right)}^2\cdotp \frac{v}{\theta^2} $$

Therefore, *RIS* emerges as the sample size estimated for a single trial with corresponding power to detect or reject an anticipated intervention effect *μ*, multiplied with a factor adjusting for the final expected or the present diversity (*D* ^*2*^) in the meta-analysis. P_C_ can be estimated as the pooled unweighted proportion of outcomes in the control groups in the included trials [11, 39–41].

Alternatively, but with equal result, the *RIS* for a random-effects model can be calculated as:

$$ R I S=4\cdotp {\left({Z_{1-}}_{\alpha /2}+{Z}_{\beta}\right)}^2\cdotp \frac{Vrandom}{\theta^2} $$

Where *ν*_*random*_ is the variance in the random-effects model. This is the model variance based calculation of *RIS*.

When the required information size has been estimated, the meta-analysis can be analysed in relation to the trial sequential monitoring boundaries, constructed as per Lan-DeMets’ critical monitoring boundaries, analogous to the sequential analysis of a single randomised trial [17, 42]. Similar to the *Z*_*i*_-value for the *i*-th cumulative meta-analysis, we use here the ratio between the logarithm of the Mantel-Haenszels weighted relative risik (*RR*_*iMH*_) and its standard error *SE*[*ln*(*RR*_*iMH*_)] in a random-effects model, e.g., as suggested by DerSimonian and Laird [44, 45]:

$$ {Z}_i = \frac{Ln\left( R{R}_{i MH}\right)}{SE\left[ Ln\left( R{R}_{i MH}\right)\right]} $$

### The required information size and the number of required extra trials in a meta-analysis

As per Elena Kulinskaya and John Wood [43], using the same mathematical notation, the sample size of future *K* ^'^ equally sized trials with equally sized (*n*_*i*_) intervention and control groups is:

1 $$ 2\cdotp {n}_i=\frac{4\cdotp {\sigma}^2\ }{\frac{K^{\hbox{'}}}{c}-{\uptau}^2}, $$

where *σ* ^2^ is the variance in these future trials and *τ* ^2^ the between trial variance in the first *K* trials, and *c* is a constant when the results from previous trials in the random-effects meta-analysis are known being:

$$ c={\left(\left[{Z}_{1-\upalpha /2}+{Z}_{1-\upbeta}\right]/\uptheta \right)}^2 - {V}_{R(K)}^{-1}, $$

*V*_*R*(*K*)_^− 1^ is the reciprocal of the variance in the random-effects model of the pooled estimate from the first *K* trials which is equal to *SD*_*R*(*K*)_^− 2^ in a meta-analysis of a continuous outcome. In the examples, given by Kulinskaya and Wood, *σ* ^2^ is determined by the simple unweighted average of the within trial variances in the first *K* trials [43]. However, as it may be more appropriate to use an estimate of the variance in the future trials which is the weighted average of the pooled squared standard deviations from the fixed-effect model, *SD*_*F*(*K*)_^2^, of the first *K* trials for continuous outcomes, we propose:

$$ {\sigma}^2= S{D}_{F(K)}^2 = \frac{N\cdot S{E}_{F(K)}^2\kern0.5em }{4}, $$

where *N* is the total number of participants in the meta-analysis of the first K trials and *SE*_*F*(*K*)_^2^ is the squared pooled standard error in the fixed-effect model. If *N* ^*’*^ is the sum of sample sizes from the equally sized *K* ^'^ future trials, the formula (1) for the sample size in future trials can be rewritten to:

$$ \frac{N^{{\textstyle \hbox{'}}}}{K^{{\textstyle \hbox{'}}}}=\frac{4\cdot {\sigma}^2}{\frac{K^{{\textstyle \hbox{'}}}}{c}-{\tau}^2}=\frac{4\cdot {\sigma}^2\cdot c}{K^{{\textstyle \hbox{'}}}-\mathrm{c}\cdot {\tau}^2}\kern1em \mathrm{and}\mathrm{thereby}\kern0.5em {N}^{{\textstyle \hbox{'}}}=\frac{4\cdot {\sigma}^2\cdot c}{1-\frac{c\cdot {\tau}^2}{K^{{\textstyle \hbox{'}}}}}\kern0.5em , $$

from which it is evident that *K’* has to be an integer greater than *c* ⋅ *τ* ^2^ (the minimum required number of extra trials) and that *N’* converges to 4 · *σ* ^2^ ⋅ *c* when *K’* approaches the infinite as:

$$ \underset{K^{{\textstyle \hbox{'}}}\to \infty }{\mathrm{Lim}\kern0.75em }\left(\frac{4\cdot {\sigma}^2\cdot c}{1-\frac{c\cdot {\tau}^2}{K^{{\textstyle \hbox{'}}}}}\right)=4\cdot {\sigma}^2\cdot c=4\cdot S{D}_{F(K)}^2\cdot c, $$

meaning that *N’* can never be less than 4 · *σ* ^2^ · *c*, despite the number of future trials being carried out. Because the random-effects model variance of the first *K* trials is always greater than the fixed-effect model variance of the *K* first trials, we have:

$$ S{D}_{R(K)}^2\ge\ S{D}_{F(K)}^2 $$

$$ 4\cdot S{D}_{R(K)}^2\cdot c>4\cdot S{D}_{F(K)}^2\cdot c, $$

and as: *c* = ([*Z*_1 − α/2_ + *Z*_1 − β_]/θ)^2^ − *SD*_*R*(*K*)_^− 2^, we get

2 $$ 4\cdot S{D}_{R(K)}^2\cdot \left\{{\left(\left[{Z}_{1-\upalpha /2}+{Z}_{1-\upbeta}\right]/\uptheta \right)}^2 - S{D}_{R(K)}^{-2}\right\}>4\cdot S{D}_{F(K)}^2\cdot c=4\cdotp {\sigma}^2\cdot c $$

If *RIS* is defined as the required information size for a meta-analysis to have as many participants as an equally powered trial to address a hypothesis of the same intervention effect. with the random-effects model variance experienced [11] so far, we will have:

$$ R I S = 4\cdot S{D}_{R(K)}^2\cdot {\left(\left[{Z}_{1-\upalpha /2}+{Z}_{1-\upbeta}\right]/\uptheta \right)}^2, $$

$$ 4\cdot S{D}_{R(K)}^2\cdot \left\{{\left(\left[{Z}_{1-\upalpha /2}+{Z}_{1-\upbeta}\right]/\uptheta \right)}^2- S{D}_{R(K)}^{-2}\right\}=4\cdot S{D}_{R(K)}^2\cdot {\left(\left[{Z}_{1-\upalpha /2}+{Z}_{1-\upbeta}\right]/\uptheta \right)}^2-4 = R I S\mathit{\hbox{-}} 4, $$

and thereby the equation (2) can be rewritten as:

$$ R I S> R I S - 4>4\cdot S{D}_{F(K)}^2\cdot c=4\cdotp {\sigma}^2\cdot c, $$

which shows that *RIS* is always greater than the minimum required extra participants in the final meta-analysis. If *V*_*R*(*K*)_^− 1^ = *SD*_*R*(*K*)_^− 2^ is small (close to zero when abundant information has already been collected), the statement that *RIS* > 4 · *σ* ^2^ · *c* is merely the trivial that the required information size will be greater than the sample size in one future trial with an anticipated variance of *σ* ^2^.

Two scenarios now cover all possible situations:

A) If: *RIS* − *AIS* > 4 ⋅ *SD*_*F*(*K*)_^2^ ⋅ *c*, then we will be able to calculate the corresponding required number (*K’*_*RIS-AIS*_) of future trials to cover the information gap indicated by the *RIS-AIS* participants according to formula (1), having:
  $$ K{'}_{RIS- AIS} = \frac{c\cdot {\tau}^2}{1-\frac{4\cdot {\sigma}^2\cdot c}{\left( RIS- AIS\right)}} $$
B) If *RIS* − *AIS* ≤ 4 ⋅ *SD*_*F*(*K*)_^2^ ⋅ *c*, then the situation is that the extra required information size is less than what is required in one extra trial. The question of whether there is an intervention effect greater or equal to θ may be answered if *RIS* has been achieved in the minimal number of required trials.

Moreover, if for example:

$$ R I S- A I S>3\cdot 4\cdot {\sigma}^2\cdot c $$

with *RIS* − *AIS* = 3 ⋅ 4 ⋅ *σ* ^2^ ⋅ *c* in the formula for *K* ’ _*RIS* − *AIS*_, then this leads to:

$$ K{'}_{12\cdot {\sigma}^2\cdot c} = \frac{c\cdot {\tau}^2}{1-\frac{4\cdot {\sigma}^2\cdot c}{3\cdot 4\cdot {\sigma}^2\cdot c}} $$

and we have:

$$ K{'}_{RIS- AIS} = \frac{3\cdot c\cdot {\tau}^2}{2}=1.5\cdot c\cdot {\tau}^2 $$

meaning that the extra number of required trials, *K* ’ _*RIS* − *AIS*_, for the random-effects meta-analysis to be adequately powered using *RIS-AIS,* would be 50% higher than the minimal required number of trials. In contrast, the minimal required number of trials, *c* ⋅ *τ* ^2^, demands a very large total information size.

### The law of reverse implication in mathematical logic

If A and B are statements that can be determined to be true or false, it follows from mathematical logic that the statement: (from *A* follows *B*), is equivalent to the statement: (from non-*B* follows non-*A*). That is: the statement (»from the null hypothesis being true follows that the result of the trial is unlikely«), is equivalent with the statement: (»from the trial result being likely follows that the null hypothesis is false«). If the probability that the trial result has emerged from a true null hypothesis is regarded as »unlikely«, when the *P*-value is lower than a certain level of significance, the null hypothesis can be rejected.

### The statistical concepts used in this article

The null hypothesis (*H*_0_) is the hypothesis that the mean or the occurence of a certain outcome is precisely the same in the groups compared.

The alternative hypothesis (*H*_A_) is the assumed magnitude of the difference between the means or the occurence of a certain outcome in the groups compared.

The *P*-value is the probability (*P*) that a specific dataset (*D*), or something even more extreme, would appear if the null hypothesis is true, that is: *P* = *P*(*D*|*H*_0_) which shold be read as: the *P*-value is equal to the probability of getting the dataset, given the null hypothesis is true.

The *P*-value is intuitively difficult to understand and is often wrongly interpreted. In Bayesian statistic, one can obtain results much easier to understand, and the clinical significance of a result from a trial or a meta-analysis may, therefore, be easier to achieve. One can present, e.g., the »reverse« probability, being how large is the probability that the null hypothesis is true given a specific dataset (*D*) from a trial or a meta-analysis: *P*(*H*_0_|*D*). Furthermore, how large is the probability that an alternative hypothesis is true given a specific dataset from a trial or a meta-analysis can be expressed as the ‘alternative’ *P*-value: *P*(*H*_A_|*D*).

Bayes factor (*BF*) is the ratio between the *P*-value and the alternative *P*-value: *BF* = *P*(*D|H*_*0*_)*/P*(*D|H*_*A*_).

Due to Bayes theorem, we have:

$$ P\left({H}_0\Big| D\right)= P\left( D\Big|{H}_0\right)\cdot P\left({H}_0\right)/ P( D). $$

A cumulative meta-analysis is a meta-analysis where the result data from the last conducted trial are added to the result data from all the previous trials and tested on the accumulated number of participants.

The *Z*-value is the general name for the test statistics, e.g., the *t*-value in a student’s *t-*test or a *χ* ^2^-test statistic in a *χ* ^2^-test or the Mantel-Haenszels test statistic. When the theoretical distribution of the test statistic is known, the *Z*-value can be converted into a *P*-value.

A cumulative *Z*_*i*_-value is the test statistic calculated after addition of the data from the *i*-th trial.

## Abbreviations

*ϴ*: Intervention effect
*α*: Maximally allowed type 1 error
*AIS*: Acquired information size in a meta-analysis
*β*: Maximally allowed type 2 error
*BF*: Bayes factor
*D*^*2*^: Statistical diversity (D-square)
*H*_*0*_: Null hypothesis
*H*_*A*_: Alternative hypothesis
*I*^*2*^: Statistical inconsistency (I-square)
*Ln*: Natural logarithm
*P*(*D|H*_*0*_): Probability of getting dataset if the null hypothesis is true
*RIS*: Required information size in a meta-analysis
*RR*: Relative risk
*SD*: Standard deviation
*SE*: Standard error
*SS*: Sample size
TSA: Trial sequential analysis
TTM Trial: Target Temperature Management Trial
*Z*_*i*_: Cumulative z_*i*_-statistics
